# Crucial mutation in the exoribonuclease domain of nsp14 of PEDV leads to high genetic instability during viral replication

**DOI:** 10.1186/s13578-021-00598-1

**Published:** 2021-06-07

**Authors:** Xiaoyu Niu, Fanzhi Kong, Yixuan J. Hou, Qiuhong Wang

**Affiliations:** 1 Center for Food Animal Health, Department of Animal Sciences, College of Food, Agricultural and Environmental Sciences, The Ohio State University, Wooster, OH 44691 USA; 2 Department of Veterinary Preventive Medicine, College of Veterinary Medicine, The Ohio State University, Columbus, OH 43210 USA; 3 College of Animal Science and Veterinary Medicine, Heilongjiang Bayi Agricultural University, No. 5 Xinfeng Road, Sartu District, Daqing, 163319 China; 4 Department of Epidemiology, University of North Carolina At Chapel Hill, Chapel Hill, NC 27516 USA

**Keywords:** Porcine epidemic diarrhea virus, Coronavirus, Nsp14, Exoribonuclease, Reverse genetics, Infectious clone

## Abstract

**Background:**

Coronavirus (CoV) nonstructural protein 14 (nsp14) has exoribonuclease (ExoN) activity, responsible for proofreading and contributing to replication fidelity. It has been reported that CoVs exhibit variable sensitivity to nsp14-ExoN deficiency. Betacoronavirus murine hepatitis virus (MHV) and severe acute respiratory syndrome (SARS)-CoV were viable upon nsp14-ExoN deficiency. While betacoronavirus Middle East respiratory syndrome (MERS)-CoV and SARS-CoV-2 were non-viable with disabled nsp14-ExoN. In this study, we investigated the nsp14-ExoN deficiency of alphacoronavirus porcine epidemic diarrhea virus (PEDV) in viral pathogenesis using reverse genetics.

**Results:**

Eight nsp14-ExoN deficient mutants, targeting the predicted active sites and the Zinc finger or mental-coordinating sites, of PEDV were designed. Only one mutant E191A with a mutation in the Mg^2+^-binding site was rescued using the infectious clone of PEDV PC22A strain (icPC22A). The passage no.1–3 (P1-3) of E191A grew to very low titers in Vero cells. To evaluate the pathogenesis of the E191A, 4 or 5-day-old gnotobiotic pigs were inoculated orally with 100 TCID_50_/pig of the E191A-P1, icPC22A, or mock. All mock pigs did not shed virus in feces or show clinical signs. All pigs inoculated with icPC22A shed high viral RNA levels, had severe diarrhea, and died by 6 days post-inoculation (dpi). In contrast, only 3 pigs (3/4, 75%) in the E191A-P1 group shed low levels of viral RNA and 2 pigs had moderate diarrhea at acute infection phase. At 22 dpi, each pig was challenged orally with 10^6^ plaque forming unit of virulent icPC22A. All pigs in the mock group developed severe diarrhea and 2 of the 5 pigs died. Pigs in the E191A-P1 group had less severe diarrhea and no pigs died. Sanger sequencing analysis revealed that the viral genome in the fecal sample of one E191A-P1-inoculated pig and the P4 virus passaged in vitro lost the E191A mutation, suggesting the genetic instability of the E191A mutant.

**Conclusion:**

The recombinant PEDV variants carrying mutations at the essential functional sites within nsp14-ExoN were either lethal or genetically unstable. Our finding further confirmed the critical role of nsp14-ExoN in CoV life cycle, suggesting that it may be a target for the design of universal anti-CoV drugs.

**Supplementary Information:**

The online version contains supplementary material available at 10.1186/s13578-021-00598-1.

## Background

Porcine epidemic diarrhea virus (PEDV) causes porcine epidemic diarrhea (PED), which is characterized by severe diarrhea and vomiting, leading to dehydration and death in neonatal pigs [1, 2]. Since 2010 when the highly virulent PEDV emerged in China, it has attacked the swine industry of many countries [2, 3]. It emerged in the United States in 2013 and caused the death of 7 million pigs during the first year of outbreaks, leading to $0.9 to $1.8 billion in economic losses to American swine industry and posing tremendous hardship for many pork producers [4, 5]. PEDV belongs to *Alphacoronavirus* genus in the family of *Coronaviridae*. It is an enveloped virus with a single-stranded, positive sense RNA genome. PEDV genome is 5’-capped, 3’-polyadenylated and consists of seven open reading frames (ORFs): ORF1a, ORF1b, and spike (S) glycoprotein, ORF3, envelope (E) protein, membrane (M) protein, and nucleocapsid (N) protein. Upon infection, ORF1a and ORF1b of viral genomic RNA are directly translated into two polyprotein precursors, pp1a and pp1ab, respectively, via a frame shift mechanism. These polyproteins are then processed into 16 nonstructural proteins (nsp1 to nsp16) carrying various functions in viral life cycles. For examples, nsp12 is an RNA-dependent RNA polymerase (RdRp) coordinating with primases, nsp7 and nsp8. Complex of nsp12-nsp7-nsp8 was defined as the minimal core component mediating coronavirus RNA synthesis [6–9]. The N-terminal domain of nsp14 is an endoribonuclease (ExoN) and is responsible for proofreading during viral RNA replication. The C-terminal domain of nsp14 is a N7-methyltransferase (N7-MTase), which cooperates with nsp16, a 2′-O-methyltransferase (2′-O-MTase), and nsp10, to perform the 5’ cap methylation of viral RNA genome to enable viral evasion of host innate immunity [10–14].

Due to the lack of ExoN proofreading activity displayed by RdRp, most RNA viruses undergo high error rates during its genome replication [15, 16]. Sanjuán et al. proposed that RNA viruses replicate in mutation rates at 10^–6^ to 10^–4^ substitution per nucleotide per cell infection (s/n/c) [17]. It was argued that this high mutation rate might be advantageous for its role in helping viruses overcome evolutionary hurdles, including escape from antiviral drugs and host immunity [17, 18]. However, vast majority of mutations showed deleterious phenotypic effect to viruses [19], suggesting that there should be a balance between favorable adaptive capacity and relatively faithful replication. Thus, the genome size of RNA virus is theoretically restricted to around ~ 15 k nucleotides [20]. However, CoVs with genome sizes up to 32 k nucleotides are exempt from that because of the proofreading activity of nsp14-ExoN. It serves as an “inspector” by checking and fixing mis-incorporated nucleotides introduced by RdRp during RNA elongation and is crucial for replication fidelity [13, 21, 22].

**Fig. 1:**
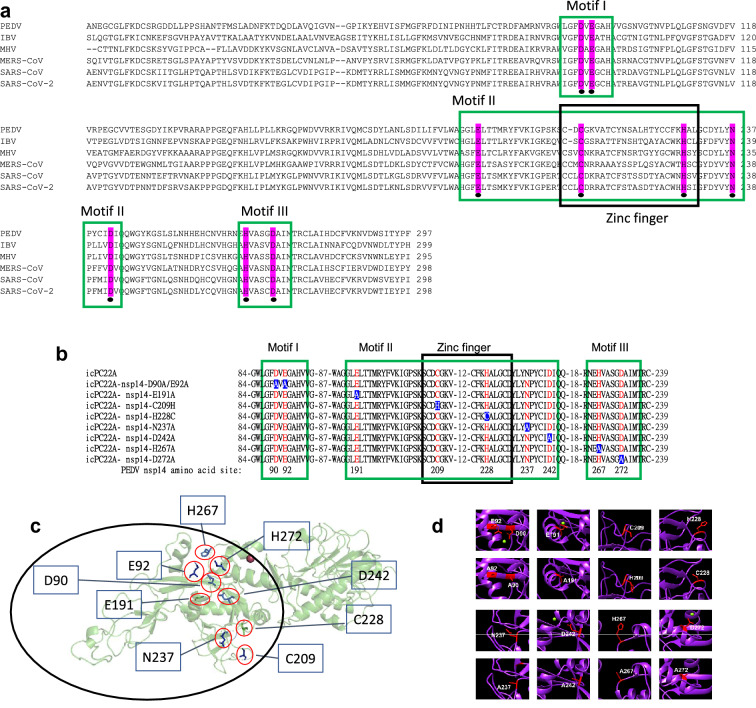
Design of recombinant PEDVs with mutations at different active sites of nsp14-ExoN. **a** Alignment of nsp14-ExoN amino acid sequences from selected coronaviruses. Sequences of the ExoN domains in PEDV (KY499262), IBV (NP_040829), MHV (NP_045298), MERS-CoV (NC-019843), SARS-CoV (NC_004718), and SARS-CoV-2 (NC_045512.2) were used for the analysis. **b** Alignment of partial nsp14 amino acid sequences of the infectious clone derived PC22A (icPC22A) and the eight PEDV nsp14 mutants designed in this study. Motifs I, II, and III conferring the ExoN active site are shown in green boxes. The Zinc fingers region is indicated in black box. The metal ion cooperating residues (D90, E92, E191, and D272), Zinc finger-related sites (C209 and H228), as well as other active sites involved in the study (N237, D242, and H267) are labeled in red. The introduced mutations are shown in blue. The numbers in gray indicate how many residues in PEDV-nsp14 are not shown. **c** 3D structural models for icPC22A nsp14. ExoN domain is circled. The metal ion cooperating residues (D90, E92, E191, and D272), Zinc finger-related sites (C209 and H228), as well as other active sites involved in the study (N237, D242, and H267) are labeled in red circles. **d** The predicted structural models for each mutation site and surrounding amino acids of wild type nsp14 and the mutants designed in this study

The nsp14-ExoN has been well defined as a member of DEDDh exonuclease family with four critical metal-coordinating amino acids in three conserved motifs, ExoN I (DE), II (E), and III (D) (Fig. 1). Metal ions Mg^2+^ or Mn^2+^ is crucial for the nsp14-ExoN nucleotides excision activity [13, 23, 24]. Alanine substitution of motif I critical residues (DE-AA) impaired ExoN activity in SARS-CoV and human common cold CoV 229E (hCoV-229E) [13, 21, 23]. Significantly higher mutation rates and decreased peak infectious titers were observed in the ExoN disrupted SARS-CoV, compared with the wildtype virus [25, 26]. Case et al. [27] confirmed that the mutation within the nsp14-ExoN motif III (D272A) of MHV made the virus susceptible to lethal mutagenesis, and the mutant showed increased sensitivity to IFN-β pretreatment. Although few studies reported the CoV nsp14 with mutated motif II based on live viruses, Minskaia et al. suggested that SARS-CoV nsp14-ExoN with D242A mutation in motif II exhibited poor nucleolytic activity compared with wildtype in tube [23]. Besides metal-coordinating residues, N237 in SARS-CoV nsp14-ExoN motif II and H267 in hCoV-229E nsp14-ExoN motif III were also active sites for nucleotide excision [23].

Zinc fingers are small, independently folded domains containing conserved cysteine and histidine residues that can coordinate with zinc^2+^, serving as the RNA or DNA recognition and binding domains. It was observed in several viral proteins with RNA binding activity, such as human immunodeficiency virus (HIV)-1 nucleocapsid, reovirus σ3, and CoV nsp14 [28–30]. Tanchou et al. [31] showed that either deletion or mutation of the Zinc finger domain in HIV-1 nucleocapsid protein led to formation of virions with abnormal core morphology and noninfectious virus particles. As for CoVs, a recombinant transmissible gastroenteritis virus (TGEV) with disrupted Zinc finger within nsp14-ExoN exhibited smaller plaques and delayed genome RNA accumulation compared with the wildtype virus [30].

Sequence alignment illustrates that the nsp14-ExoN of CoVs across different genera are highly conserved (Fig. 1a). We hypothesized that recombinant PEDVs with deficient nsp14-ExoN are significantly attenuated. To test this hypothesis, we designed eight mutants targeting nsp14-ExoN active sites, metal-coordinating sites, and Zinc fingers. One mutant was rescued and characterized in Vero cells. Furthermore, we examined the pathogenicity of this mutant in neonatal gnotobiotic (Gn) pigs.

## Results

### One recombinant PEDV with deficient nsp14 was rescued

Parental PEDV nsp14 and its counterparts with deficient ExoN were designed and their structures were modelled using SWISS-MODEL with template 5C8S.1.B (Fig. 1a, b) [32]. Based on our structural analysis, the putative catalytic sites will be diminished upon mutagenesis in all designed mutants (Fig. 1c). All the eight designed recombinant PEDVs were subjected to virus recovery in Vero cells using an infectious cDNA clone of virulent PEDV stain PC22A (icPC22A) described previously [33]. Among those mutants, only the recombinant PEDV carrying E191A mutation was viable. Supernatants containing rescued viruses were harvested, designated as the P0 of E191A mutant. One clone of the mutant was selected by plaque assay and passaged once to generate the P1 virus stock. The complete genome of E191A-P1 was verified by Sanger sequencing.

### Low passages of E191A mutant showed poor replication in vitro

**Fig. 2:**
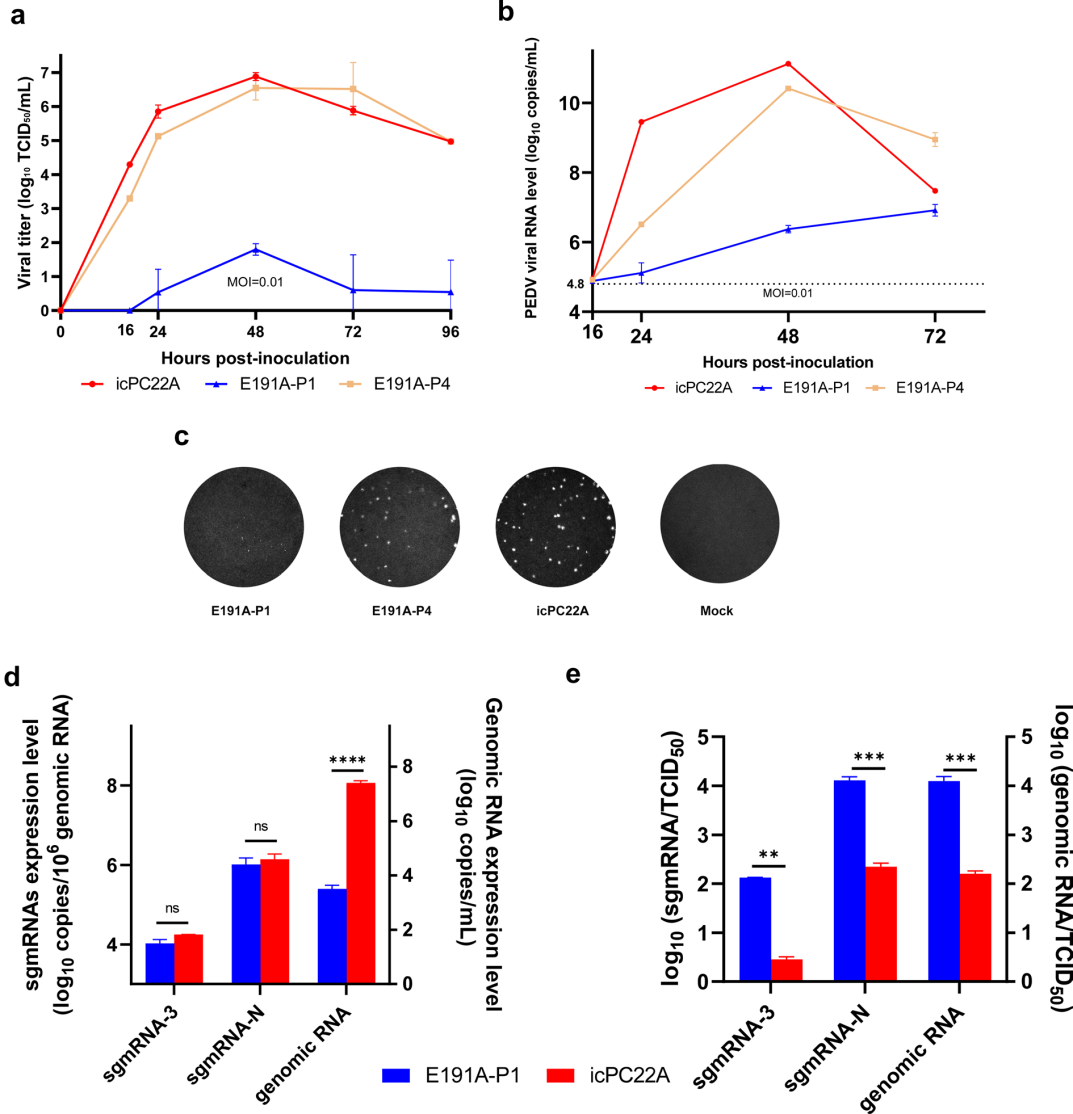
In vitro Characterization of the recombinant nsp14-ExoN mutant E191A. **a**, **b** Multi-step growth curves of recombinant PEDVs (E191A-P1, E191A-P4, and the parental icPC22A) in Vero cells using an MOI of 0.01. Due to the very low infectious titers of E191A-P1, the RNA titers presented in **b** was employed to interpret the growth kinetics of recombinant PEDVs; **c** plaques of recombinant PEDVs in Vero cells overlayed with 1.5% agarose. The cells were fixed and stained with crystal violet at 72 hpi. **d**, **e** Vero cells infected with recombinant PEDVs at a MOI of 0.01 was harvested at 24 hpi. The expression level of sgmRNA-3 (left Y axis), sgmRNA-N (left Y axis), and genomic RNA (right Y axis) were presented in **d** and the sgmRNAs/TCID_50_ (left Y axis) or genomic RNA/TCID_50_ (right Y axis) ratios were shown in **e**. **, *p* < 0.01; ***, *p* < 0.005; ****, *p* < 0.001; ns, no significance

Low passage (P1-P3) of E191A mutant replicated to much lower infectious titers (1.80 ± 0.12 log_10_ TCID_50_/mL) than icPC22A (5.14 ± 0.23 log_10_ TCID_50_/mL) in Vero cells, which is an efficacious PEDV propagation system [34]. In addition to Vero cells, porcine intestinal epithelial cells, IPEC-DQ, was also used for the propagation of E191A mutant [35]. However, the low passages of the recombinant virus displayed poor replication in both cell lines. It showed peak infectious titers of 1.42 ± 0.16 log_10_ TCID_50_/mL in IPEC-DQ cells. The E191A-P4 exhibited dramatically high infectious titer (5.55 ± 0.35 log_10_ TCID_50_/mL), which was not significantly different from icPC22A (*p* = 0.3002) in Vero cells (Fig. 2a, b). The E191A-P1 showed much smaller plaques (0.2268 ± 0.0790 mm in diameter) than the P4 virus (0.5236 ± 0.1537 mm in diameter), which was similar to icPC22A (0.4412 ± 0.1510 mm in diameter) (Fig. 2c). To analyze the effect of E191A mutation on PEDV subgenomic mRNAs (sgmRNAs) and genomic RNA synthesis, we analyzed sgmRNA-3 and sgmRNA-N. Vero cells inoculated with either E191A-P1 or icPC22A were harvest at 24 hpi and subjected to qRT-PCR detection. Similar levels of sgmRNA-3 and sgmRNA-N were observed in E191A mutant and icPC22A, but the mutant showed significantly lower genomic RNA titers at 24 hpi than icPC22A (Fig. 2d). We calculated the sgmRNAs/TCID_50_ and genomic RNA/TCID_50_ ratios and found that E191A had significantly higher ratios than icPC22A. These results suggest dramatically reduced replication efficiency of E191A mutant (Fig. 2e).

### E191A-P1 was attenuated in neonatal piglets

**Fig. 3:**
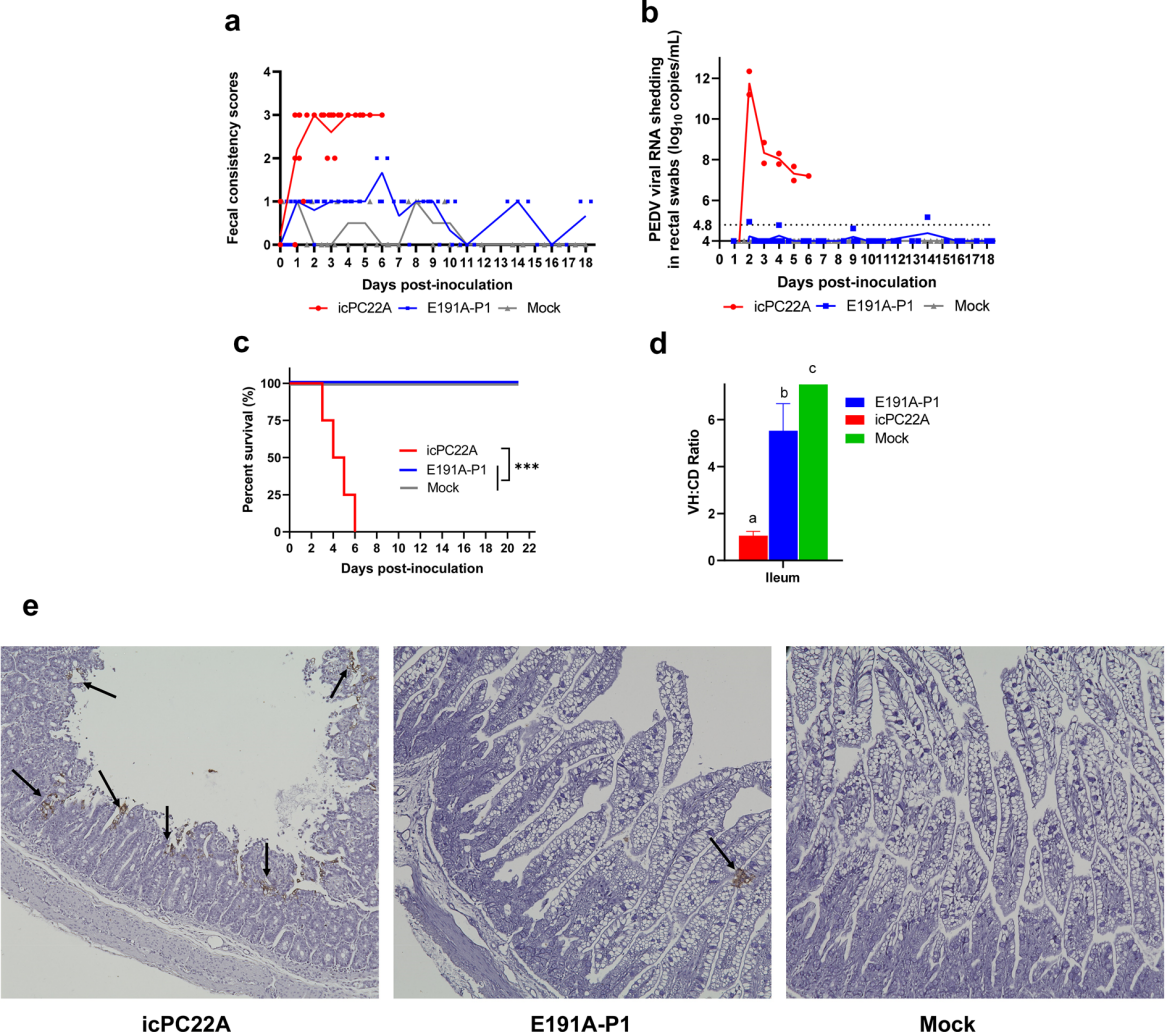
Pathogenicity of the recombinant PEDVs in Gn piglets. **a** Fecal consistency scores of pigs within 18 dpi. Fecal consistency was scored as follows: 0, solid; 1, pasty; 2, semiliquid; and 3, liquid. Scores of ≥ 2 and 3 were considered diarrhea and severe diarrhea, respectively. Each dot represents the score of an individual pig; each line indicates the mean scores of a group. Pigs with fecal scores greater than 2 were defined as having diarrhea and a score of 3 was severe diarrhea. All pigs in the icPC22A group died by 6 dpi. **b** PEDV RNA (N gene) shedding titers in rectal swabs within 18 dpi. Each symbol represents the titer of an individual piglet; each line indicates the mean values of a group. The dash line at 4.8 log_10_ copies/mL indicates the detection limit. **c** Survival curves of pigs after inoculation by 21dpi. **d** Villous height to crypt depth (VH: CD) ratios of ileum of piglets euthanized at 3 dpi. Groups with significant differences are indicated with different letters. **e** Immunohistochemistry staining of PEDV N proteins in the enterocytes of ileum of piglets (magnification, 100 ×). The brown signals, indicated by arrows, represented the PEDV antigens in enterocytes and were observed in the icPC22A- and E191A-P1-inoculated, but not in mock piglets

Neonatal Gn pigs were used to investigate pathogenesis and immunogenicity of the E191A mutant. The icPC22A-inoculated pigs (5/5) developed severe diarrhea from 2 dpi and died within 6 dpi. In contrast, only two pigs showed moderate diarrhea at 6 dpi and no mortality was observed in E191A-P1 group (Fig. 3a, c). The virulent icPC22A-inoculated pigs shed high levels of PEDV RNA titers (12.12 ± 0.53 log_10_ copies/mL) in feces from 1.20 ± 0.40 dpi (Fig. 3b, Table 1). In comparison, significantly diminished and delayed PEDV fecal RNA shedding (5.06 ± 0.16 log_10_ copies/mL) was detected in three E191A-P1-inoculated pigs (Fig. 3b, Table 1). By macroscopic examination, the intestines of pigs from icPC22A group exhibited typical PEDV lesions, characterized by thin and transparent intestinal walls. Large amounts of yellowish fluid were found in the intestinal lumen. However, no lesions were observed from the E191A-P1-inoculated pigs. All mock pigs remained healthy. Immunohistochemical (IHC) staining of pig intestine was performed to confirm virus infection and show histopathological changes. Villous height: crypt depth (VH: CD) ratios of villi was used to describe the severity of villous atrophy of the infected pigs [36]. Histopathological analysis indicated that severe villous atrophy was caused by virulent icPC22A infection as reported previously [36], minor microlesion was observed in E191A-P1-inoculated pigs, but not in the mock pigs (Fig. 3d). Robust icPC22A replication was verified by IHC staining of PEDV N proteins in ileum (Fig. 3e). However, only sporadic N protein staining was observed in ileum of pigs infected with E191A-P1.

**Table 1:** Summary of clinical signs and PEDV shedding of Gn piglets after PEDV inoculation (1–21 dpi)

Group	No. of pigs	Mortality rate	Diarrhea rate	Onset of diarrhea (dpi)	Peak mean RNA shedding titer (log_10_ copies/mL)	Onset of peak RNA shedding (dpi)
icPC22A	6 a	100% (5/5) b	100% (5/5) b	1.20 ± 0.40 b	12.12 ± 0.53 b	1.20 ± 0.40
E191A-P1	4 a	0 (0/3) c	66.7% (2/3) c	6.00 ± 0.00 c	5.06 ± 0.11 c	6.67 ± 5.25
Mock	6 a	0 (0/5)	0 (0/5)	NA	NA	NA

^a^One pig from each group were euthanized at 3 dpi for histopathological examination and were excluded for the calculation of Mortality and Diarrhea rates, Peak mean RNA shedding and Onset of peak RNA shedding

*dpi* days post-inoculation

Fecal scores of 2 and 3 were considered as moderate diarrhea and severe diarrhea, respectively

^b,c^Different letters denote significant difference between groups (*p* < 0.05)

*NA* not available

### E191A mutant induced partial protection against virulent icPC22A challenge in pigs

**Fig. 4:**
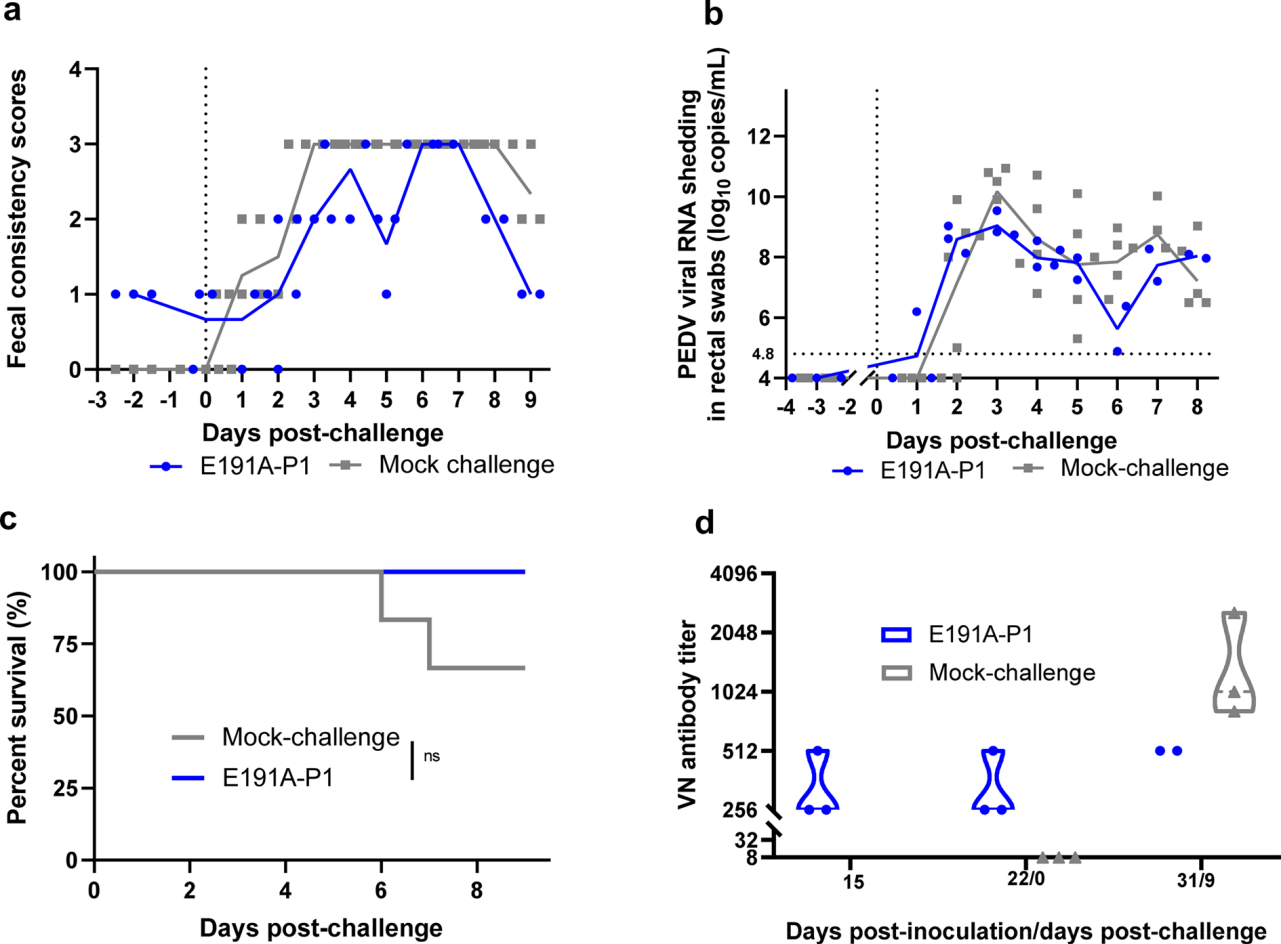
Induction of partial protection by E191A-P1 mutant in Gn pigs against icPC22A challenge. **a** Fecal consistency scores of pigs post challenge. Fecal consistency was scored as follows: 0, solid; 1, pasty; 2, semiliquid; and 3, liquid. Scores of ≥ 2 and 3 were considered diarrhea and severe diarrhea, respectively. Each dot represents the score of an individual pig; each line indicates the mean scores of a group. A pig with a score of ≥ 2 was defined as having diarrhea and a score of 3 was severe diarrhea. **b** Fecal PEDV RNA shedding profile of pigs post challenge. Each line indicates the mean values of a group. The dash line at 4.8 log_10_ copies/mL indicates the detection limit. **c** Survival curves of pigs by 9 dpc. **d** VN antibody titers in serum samples collected at different time points

At 22 dpi, pigs in the E191A-P1 and mock groups were challenged with the highly virulent icPC22A at a high dose (10^6^ PFU/pig). All E191A-P1-inoculated pigs had moderate-severe diarrhea and the accumulated days for diarrhea was 2.33 ± 0.58 days (Fig. 4a, Table 2). However, all mock-challenged pigs showed severe diarrhea and lasted for 6.33 ± 0.47 days (Fig. 4a, Table 2). Two of five pigs in the mock group died at 6- or 7- days post-challenge (dpc), while no mortality was observed in the E191A-P1 group (Fig. 4c). Pigs in E191A-P1 group exhibited peak PEDV RNA shedding (9.03 ± 0.44 log_10_ copies/mL) at 3 dpc (Fig. 4b, Table 2), whereas pigs in the mock-challenge group shed a significantly higher peak viral RNA level (10.43 ± 0.87 log_10_ copies/mL) (*p* = 0.0010) at 3 dpc. The serum viral neutralizing (VN) antibodies targeting PEDV were tested using microneutralization assay based on reduction of TCID_50_. VN antibodies were elicited at 15 dpi upon E191A-P1 mutant inoculation. Significantly higher serum VN antibody titers were detected in mock-challenged pigs than the E191A-P1-inoculated pigs at 31 dpi/9 dpc (*p* = 0.2000) (Fig. 4d). In summary, E191A-P1 inoculation provided pigs with partial protection, with reduced viral shedding and severity of diarrhea against challenge with the highly virulent icPC22A.

**Table 2:** Clinical signs and PEDV shedding of pigs challenged with the highly virulent icPC22A (1–9 dpc)

Group	No. of pigs	Mortality rate	Severe diarrhea rate	Onset of severe diarrhea (dpc)	Duration of severe diarrhea (days)	Duration of diarrhea (days)	Peak mean RNA shedding titer (log_10_ copies/mL)
E191A-P1	3	0 (0/3)	100% (3/3)	4.67 ± 1.15	2.33 ± 0.58 a	5.33 ± 1.15 a	9.03 ± 0.44 a
Mock	5	40% (2/5)	100% (5/5)	4.00 ± 0.00	6.33 ± 0.470 b	8.00 ± 0.00 b	10.43 ± 0.87 b

dpc: days post challenge

Fecal scores of 2 and 3 were considered as moderate diarrhea and severe diarrhea, respectively

a, b: Different letters denote significant difference between groups (*p* < 0.05)

### The Recombinant E191A mutant was genetically unstable.

**Fig. 5:**
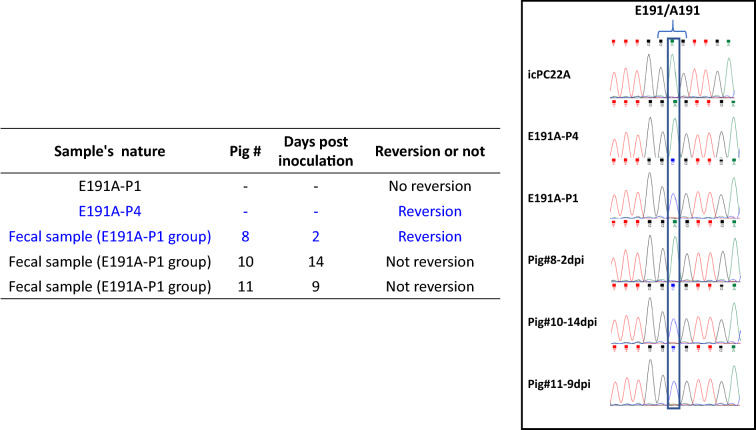
Genetic instability of E191A mutant. Sanger sequencing showed that E191A-P4 and the fecal sample collected at 2 dpi from the E191A-P1-inoculated pig#8 reverted to wildtype ExoN (GAG)

CoV nsp14 is a critical replicase and its ExoN activity is responsible for replication fidelity, absence of which may cause incredible high mutation rate [25, 26]. To evaluate genetic stability, E191A-P4 as well as the viruses shed in fecal samples of E191A-P1 inoculated pigs were subjected to Sanger sequencing. We found that E191A-P4 has lost the E191A mutation and reverted to wildtype (Fig. 5). Viral RNA copy numbers in the samples from fecal and intestinal contents of E191A-P1-inoculated pigs were too low to get the whole genome sequence. Instead, the nsp14 region was sequenced for those samples (Fig. 5). Among the four pigs inoculated with E191A-P1, the viruses from two pigs (pig#10 and #11) retained the E191A mutation and had no additional mutations within nsp14, but the virus from pig#8 had reverted to wildtype nsp14 by 2 dpi (Fig. 5). No additional mutations were detected in nsp14 from these three samples. Collectively, these data suggest that recombinant PEDV E191A quickly reverted to wildtype either in vivo or in vitro, indicating the low genetic stability of ExoN mutant.

## Discussion

PEDV infection still presents long-term challenges to the swine industry with ability to spread locally among swine farms even with sound biosecurity practice. To prevent the PEDV epidemic, U.S. Department of Agriculture has conditionally licensed two vaccines, one inactivated vaccine (Zoetis Inc.) and a Venezuelan equine encephalitis virus (VEEV) vectored vaccine (Merck & Co., Inc.), for use in sows. Previous pig challenge studies showed that both vaccines had limited efficiency when they were used for PEDV-naive sows in protecting piglets from PED [37, 38]. Lessons from protecting pigs from some other swine enteric viruses, such as rotavirus and TGEV, as well as the effective candidate presented by our lab previously [36], suggest that live attenuated vaccines (LAVs) may be a strategy for PED prevention.

CoV nsp14 plays a critical role in the viral replication event by guaranteeing the genetic fidelity via its ExoN domain. An engineered SARS-CoV with modified nsp14-ExoN showed profoundly decreased fidelity, as well as attenuation in vivo [26]. As nsp14 is highly conserved among CoVs (Fig. 1), it may be a proper target for the development of LAVs for PEDV. Despite several betacoronaviruses, MHV and SARS-CoV, with deficient nsp14-ExoN have been successfully rescued using reverse genetics [25, 26, 39], few attempts were made for alphacoronaviruses. Becares et al. have attempted to generate sets of TGEV mutants lacking ExoN activity, but no recombinant viruses were rescued [30]. There should be at least three steps for the removal of mis-incorporated nucleotides by ExoNs: (a) selection and binding to the mismatched nucleotides during replication, (b) hydrolysis of phosphodiester bonds between selected nucleotides and RNA chain by ExoNs, and (c) translocation of the mismatched nucleotide and replaced by correct one. In this study, we used PEDV as a model of alphacoronavirus to evaluate the role of nsp14 for viral pathogenesis. Eight recombinant PEDVs with inactivated nsp14 were designed and subjected for recovery, and only one bearing E191A mutation was rescued. Comparably, Ogando et al. reported that ExoN inactivation by either conservative or alanine substitutions of the catalytic residues of MERS-CoV nsp14-ExoN abolished the recovery of infectious progeny and detectable viral RNA synthesis [39]. Among all 13 mutants they designed, only the one carrying E191D mutation could be recovered. In both our and Orgando’s study, unlike those unviable mutants targeting critical catalytic sites or direct substrate recognition and binding sites, engaged in steps (a) and (b), the E191 residue of nsp14-ExoN coordinates with Mg^2+^ in nsp14-nsp10 complex structure [13]. According to the two-metal-ion mechanism for the 3′-5′ exonuclease activity [40], the E191-associated Mg^2+^ is responsible for facilitating the removal of mis-incorporated nucleotides, which is involved in the step (c). We confirmed that all mutants with abrogated ExoN activity were lethal, except one carrying the mutant affecting the release of products can be rescued. Further biochemical studies are needed to delineate detailed functions of those critical sites in removing mis-incorporated nucleotides.

The reversion of the attenuated viruses to virulent strain is a safety concern in LAV development. In our study, the recombinant PEDV carrying modified nsp14 reverted in both in vivo and in vitro environments. Low passages of the recombinant virus exhibited poor replication both in vitro and in vivo. In vitro growth curve showed that the RNA level of E191A-P1 kept increasing even the infectious titer started to drop after 48 hpi (Fig. 2b). It suggested that RNA synthesis for E191A-P1 was competent but accumulated mutations caused the deficiency in generation of infectious progeny. In the four pigs inoculated with E191A-P1 mutant, three pigs (3/4, 75%) had positive viral RNA shedding in feces, and the mutant virus in pig #8 reverted at 2 dpi by sequence analyses. Numerous selective pressures, such as host immune responses and intestinal environment, worked on PEDV replication, and differ in individual pigs. Compared with wild type PEDV, E191A-P1 with deficient proofreading activity must have a higher mutation rate. All these factors contribute to the emergence of the reverse mutation in pig#8 as early as 2 dpi, but not in pig #10 by 14 dpi and pig #11 by 9 dpi. If more pigs were tested, E191A-P1 reversion may occur at different dpi. Conversely, the P4 of that recombinant PEDV displayed distinct characteristics from the lower passages P1-P3. The whole genome sequencing of it illustrated that the P4 of E191A mutant has lost the introduced E191A mutation. Five pigs initially inoculated with E191A-P1 without viral RNA shedding in feces or diarrhea by 3 dpi were re-inoculated with E191A-P4 (100 TCID_50_/pig) to characterize the pathogenesis of E191A-P4. And all pigs showed severe diarrhea comparable to virulent icPC22A (Additional file 1: Figure S1). Although the onset of diarrhea and viral shedding were slightly delayed in E191A-P4-inoculated pigs, it may be resulted from pigs of different day ages when being inoculated. Similarly, reversion events were also observed in Orgando’s unviable mutants when trying to rescue ExoN-deficent MERS-CoVs [39]. However, results from Dr. Denison lab told another story about CoV nsp14-ExoN [40]. The MHV-ExoN(-) was engineered with disrupted ExoN activity and serially passaged for 250 passages in vitro. Compared with the low passage (P3) of that recombinant virus, MHV-ExoN(-) P250 showed significantly enhanced replication fidelity and increased competitive fitness, as well as less susceptibility to nucleoside analogues. After long-term passages, MHV-ExoN(-) reached identical growth kinetics to the wildtype MHV by P100. Although no reversion mutation was found, 171 mutations were archived throughout the whole genome, which may serve as compensatory mutations responsible for the fitness achieved by MHV-ExoN(-) P250. Both our study and data from previous reports suggested that deficient nsp14-ExoN posed selective pressure to the mutants. Confronting strengthened pressure, CoV employed alternative strategies to survive. One is hundreds of compensatory mutations which may contribute to the interplay of multiple replicase proteins to make it more viable, and the other is the reversion mutation observed in our study.

To protect from pathogen infection, a highly efficient immune system, comprising of innate immunity and adaptive immunity, is well developed in eukaryotic organisms by evolution. It has been proposed that CoV nsp14 may act as an innate immune antagonist by downregulating the RNA intermediates. Lassa virus (LASV), another RNA virus encoding ExoN domain by its nucleoprotein, employed this strategy to counteract host innate immunity. The recombinant LASV with abolished ExoN activity triggered much more robust NK cell response and more abundant IFN-γ expression [41]. In terms of CoV nsp14, previous studies revealed that, upon the infection of recombinant TGEV carrying mutation within Zinc finger, decreased accumulation of dsRNA and IFN-β were observed [30]. In our study, partial protection from severe diarrhea and viral shedding was induced by E191A-P1 infection. Even the reversion event occurred in pig#8 in E191A-P1 group, the pig did not develop watery diarrhea or high level of viral RNA shedding as virulent icPC22A inoculated pigs. Possible reasons are listed here: (1) There were potential compensate mutations throughout the viral genome of the reverted virus, which were acquired during viral replication in the pig and may contribute to this attenuated phenotype; (2) local mucosal immune response elicited by limited E191A-P1 replication at the early stage of infection help protect the pig from severe diarrhea which could be caused by the reverted virus. In terms of adaptive immunity, although replicating poorly, E191A-P1 mutant elicited VN antibody titers by 15 dpi. Both innate and adaptive immune responses may contribute to the partial protection provided by E191A-P1 infection.

Several deadly coronaviruses have emerged and caused serious problem in both humans and animals in past two decades, such as SARS-CoV, MERS-CoV, SARS-CoV-2, and genogroup 2 PEDV. The ongoing pandemic of COVID-19, caused by SARS-CoV-2, makes the development of universal strategy to control and prevent CoV infection a hot topic. There have been several viral proteins prioritized as anti-COVID-19 drug targets: the main protease (Mpro) or 3C-like protease (3CLpro), Papain-like protease (PLpro), and RdRp [42–46]. All those are crucial replicases for viral survival, replication, and transmission. Meanwhile, the catalytic pocket residues of these enzymes are highly conserved and share sequence similarity among CoVs. For example, Remdesivir is an adenosine analog inhibiting RdRp activity and has broad-spectrum antiviral function [46]. It was initially reported as a drug against MERS-CoV and then approved by Food and Drug Administration (FDA) for emergency use authorization for anti-COVID-19 therapy [47]. Since the indispensable role of CoV nsp14-ExoN in repairing mis-incorporated nucleotides and all those critical catalytic sites responsible for proofreading were conserved among CoVs, it is also a good target for universal anti-CoV drug development. Previous reports suggested that CoVs carrying deficient nsp14-ExoN are either unviable or more sensitive to nucleotide analogue [25, 39, 40]. Therefore, the combination of CoV nsp14-ExoN inhibitors and other anti-CoV drugs, such as remdesivir, may exhibit synergy against SARS-CoV-2, which can be investigated in the future. In summary, we showed that the recombinant PEDV mutant E191A with deficient nsp14-ExoN was viable, but it replicated poorly in vitro and in vivo. Meanwhile, the reversion mutation has occurred quickly both in vitro and in vivo, indicating the genetic instability of this recombinant virus. Our data demonstrated that PEDV nsp14 mutants carrying mutations at the essential functional sites within ExoN were either lethal or genetically unstable. Although it is possible to rescue viable and robust-replicating recombinant PEDVs with disrupted nsp14-ExoN by engineering additional fitness mutations, its properties of lacking proofreading and low fidelity would be detrimental to LAV development by diminishing attenuating mutations or introducing unintended mutations. In this study, we mainly focused on the effect of modified CoVs replicase nsp14-ExoN on viral pathogenesis. It would be more informative if the enzymatic activity of nsp14-ExoN of E191A-P1 mutant is characterized.

## Conclusion

We identified nsp14-ExoN as an indispensable factor in PEDV life cycle. Abolishing its function leads to either lethal or genetically unstable viruses; it is not an appropriate target for the design of PEDV LAVs but may be a target for the development of universal anti-CoV drugs.

## Materials and methods

### Cells and reagents

Vero cells (ATCC number: CCL81) were cultured in Dulbecco's modified Eagle's medium (DMEM, Life Technologies, Carlsbad, CA) in the presence of 5% fetal bovine serum (FBS, Hyclone, Logan, UT, USA), and antibiotics (100 U/ml penicillin, 100 μg/ml streptomycin, and 250 ng/ml amphotericin B, Life Technologies, Carlsbad, CA, USA). After inoculated with PEDV, Vero cells were maintained in DMEM supplemented with 0.3% tryptose phosphate broth (TPB, Life Technologies, Carlsbad, CA, USA), 100 U/ml penicillin (Life Technologies, Carlsbad, CA, USA), 100 μg/ml streptomycin (Life Technologies, Carlsbad, CA, USA), and 10 μg/ml trypsin (Life Technologies, Carlsbad, CA, USA) as described previously [34]. IPEC-DQ cells, a subline derived from the porcine intestinal epithelial cell line IPEC-J2, were kindly provided by Dr. Dongwan Yoo, the University of Illinois at Urbana-Champaign. IPEC-DQ cells were cultured in RPMI 1640 (Gibco, Carlsbad, CA) supplemented with 10% FBS and the antibiotics. After PEDV inoculation, IPEC-DQ cells were maintained in RPMI 1640 supplemented with 0.3% TPB (Life Technologies, Carlsbad, CA, USA) and 10 μg/ml trypsin as described [36].

### Structural modeling and sequence alignment

The three-dimensional structural modeling of PEDV nsp14-ExoN and the eight designed mutants were performed at SWISS-MODEL (https://swissmodel.expasy.org) using SARS-CoV nsp14 (SMTL ID: 5C8S.1.B) as the template. The structural analysis was carried out with UCSF Chimera (http://www.rbvi.ucsf.edu/chimera).

### Generation of recombinant PEDVs with mutated nsp14

Eight mutants carrying mutation targeting PEDV-nsp14-ExoN active sites, metal ion coordinating sites, and Zinc fingers were designed (Fig. 1a, c) and subjected for recovery based on the full-length cDNA clone of PEDV strain PC22A as described previously [33]. Briefly, the point mutations were introduced into the plasmid pUC19 carrying the fragment D of icPC22A via NEB Q5® Site-Directed Mutagenesis Kit (NEB, Ipswich, MA, USA). Then the insertion sequences of plasmids were confirmed by sanger sequencing. After the plasmids being digested by restriction enzymes, the appropriately sized cDNA inserts were purified using the QIAquick gel extraction kit (Qiagen, Hilden, Germany). All five fragments (A, B, C, D, and E) were ligated with T4 ligase (NEB, Ipswich, MA, USA) at 4 °C overnight in an equal molar ratio for full-length cDNA. The ligated full-length cDNAs were purified by chloroform extraction and used as templates for in vitro transcription using a mMessage mMachine T7 transcription kit (Ambion, Austin, CA, USA). The polyadenylated PEDV N gene transcript was generated with an inserted T7 promoter and co-electroporated into the Vero cells with the full-length transcripts at 450 V and 50 μF using a Gene Pulser II electroporator (Bio-Rad, Hercules, CA, USA). At 18 h after electroporation, the growth medium was discarded, and Vero cells were cultured in maintenance medium in the presence of 10 μg/ml trypsin. Cells were monitored for cytopathologic effect (CPE) and harvested, designed as P0 virus. The P0 virus was subjected to plaque purification and one clone was propagated to generate the P1 stock for the mutant. The full genome of the P1 virus stock was confirmed by sanger sequencing.

### Plaque assay

Monolayers of Vero cells in six-well plates were incubated with tenfold serially diluted icPC22A or the mutant for one hour in the presence of trypsin. After that, the inoculum was removed, and cells were washed with PBS. Then cell monolayers were covered with 2 mL/well of overlay containing 1.5% agarose in MEM supplemented with 10 μg/ml trypsin and 0.3% TPB as described previously [34]. At 3 dpi, the cells were fixed with 10% PBS-buffered formalin for 15 min and stained with 0.2% crystal violet.

### Growth kinetics for recombinant PEDVs

Vero cell monolayers in six-well plates were infected with the corresponding viruses at a multiplicity of infection (MOI) of 0.01. After the one-hour adsorption, cells were washed with PBS to remove those unbound virions and cultured in maintenance medium. The supernatants were collected at multiple time points [16-, 24-, 48-, 72-, and 96-h post inoculation (hpi)]. Supernatants were titrated in 96-well plates for 50% tissue culture infective doses (TCID_50_) by the Reed-Muench method [48].

### Design of the experimental infection of Gn pigs

All experiments carried out in this study were approved by the Institutional Animal Care and Use Committee (IACUC) of The Ohio State University. Gn pigs were delivered from PEDV-free sows as described previously [49] and divided into 3 groups. At 4–5 days of age, Gn piglets were orally inoculated with the P1 of E191A mutant (E191A-P1) (n = 9; 100 TCID_50_/pig), icPC22A (n = 6; 100 TCID_50_/pig), or mock (n = 5; DMEM), respectively. One piglet in the icPC22A group (pig#1) and one showing positive viral RNA shedding (pig#8) from E191A-P1 group were euthanized at 3 dpi. At 22 dpi, each pig was challenged orally with 6 log_10_ PFU of icPC22A and housed for one more week to evaluate protection against virulent PEDV challenge. After inoculation, clinical signs, including diarrhea and vomiting were monitored twice a day. Rectal swabs were collected daily. The severity of diarrhea was scored based on the fecal consistency (FC) in individual pigs: 0, solid; 1, pasty; 2, semiliquid (moderate diarrhea); and 3, liquid (severe diarrhea) [36, 50–53]. To determine the genetic stability of E191A mutant in pig model, some fecal samples were subjected for sanger sequencing. The viral RNA shedding in the rectal swab were determined by reverse transcription-quantitative PCR (RT-qPCR) as described previously [36].

### Immunohistochemical staining

The formalin-fixed ileum samples were processed using a non-biotin polymerized horseradish peroxidase system (BioGenex Laboratories, San Ramon, CA, USA) for IHC staining. Tissues were counterstained with hematoxylin. Monoclonal antibody SD6-29 (gift of Eric Nelson, South Dakota State University) targeting PEDV nucleocapsid (N) proteins was used as the primary antibody. Eight villi and crypts were selected and measured for VH:CD ratios for ileum of pigs in each group as described previously [36].

### RNA extraction and reverse transcription quantitative PCR (RT-qPCR)

Rectal swab samples were collected and subjected to total RNA extraction using MagMax RNA isolation kit (Austin, CA, USA) and MagMAX™ Express instrument (Thermofisher, Waltham, MA, USA) according to the manufacturer’s instructions. After that, the PEDV RNA titers of swab samples were determined by RT-qPCR using the OneStep RT-PCR Kit (QIAGEN, Valencia, CA, USA) with primers (forward 5′-TGAAGCCGTCTCATACTATTCTG-3′, and reverse 5′-AATCCCTCAACAGTGTCAGC-3′) and the probe (5′-FAM-TGCAATGCCGTTTCGTGTCCTTC-BHQ-3′) as described previously [36]. Copy numbers of each sample were determined by standard curves.

Vero cells infected with icPC22A or E191A-P1 at a MOI of 0.01 was harvested at 24hpi and subjected to total RNA extraction using TRIzol™ Reagent (Invitrogen, Carlsbad, CA). The cellular DNA was removed by treating the RNA with DNase I (NEB, Ipswich, MA, USA). 500 ng RNA was used for sgmRNAs and genomic RNA detection. sgmRNA-3 was amplified with forward primer (5′-CTATCTACGGATAGTTAGCTC), reverse primer (5′-CTGTGTCAATCGTGTATTG), and probe [6-carboxyfluorescein (FAM)- 5′-ACATCACTGCACGTGGAC- black hole quencher (BGH)]. sgmRNA-N was amplified with forward primer (5′-CTCTTGTCTACTCAATTCAACTAAACAGAAAC), reverse primer (5′-CCAGTATCCAATTTGCTGGTCC), and probe (FAM-5’-TCAGGATCGTGGCCGCAAAC-BHQ). The genomic RNA was amplified with forward primer (5’-TGAAGCCGTCTCATACTATTCTG), reverse primer (5’-AATCCCTCAACAGTGTCAGC), and probe (FAM-5’-TGCAATGCCGTTTCGTGTCCTTC-BHQ). Copy numbers of each were determined by standard curves.

### Statistical analysis

The statistical analyses were performed using GraphPad Prism 6.0. Data in Fig. 2a, b are shown in Mean ± standard deviation (SD). Figures 2d, e, and 3d were analyzed by multiple t tests. Figures 2c, 4c, d were analyzed by one-way analysis of variance (ANOVA) followed by Tukey’s multiple-comparison test.

## Supplementary Information

**Additional file 1: Figure S1.** Pathogenicity of the E191A-P4 in Gn piglets that were initially inoculated with E191A-P1 but did not shed virus by 3 dpi and re-inoculated with E191A-P4 at 3 dpi. **(A)** Fecal consistency scores of pigs after the E191A-P4 inoculation. Fecal consistency was scored as follows: 0, solid, 1, pasty, 2, semiliquid; and 3, liquid. Scores of ≥ 2 and 3 were considered diarrhea and severe diarrhea, respectively. Each dot represents the score of an individual pig; each line indicates the mean scores of a group. **(B) **PEDV RNA (N gene) shedding titers in rectal swabs after inoculation. Each line indicates the mean values of a group. The dash line at 4.8 log_10_ copies/mL indicates the detection limit. **(C) **Survival curves of pigs after inoculated with E191A-P4.

## Data Availability

The data supporting the conclusions of this article is available to all interested researchers upon request**.**
