# Supplementary figures and images for: Crucial mutation in the exoribonuclease domain of nsp14 of PEDV leads to high genetic instability during viral replication
Source: Cell Biosci. 2021 Jun 7;11:106. doi: 10.1186/s13578-021-00598-1 (PMC8182996; doi:10.1186/s13578-021-00598-1)

## Slide 1
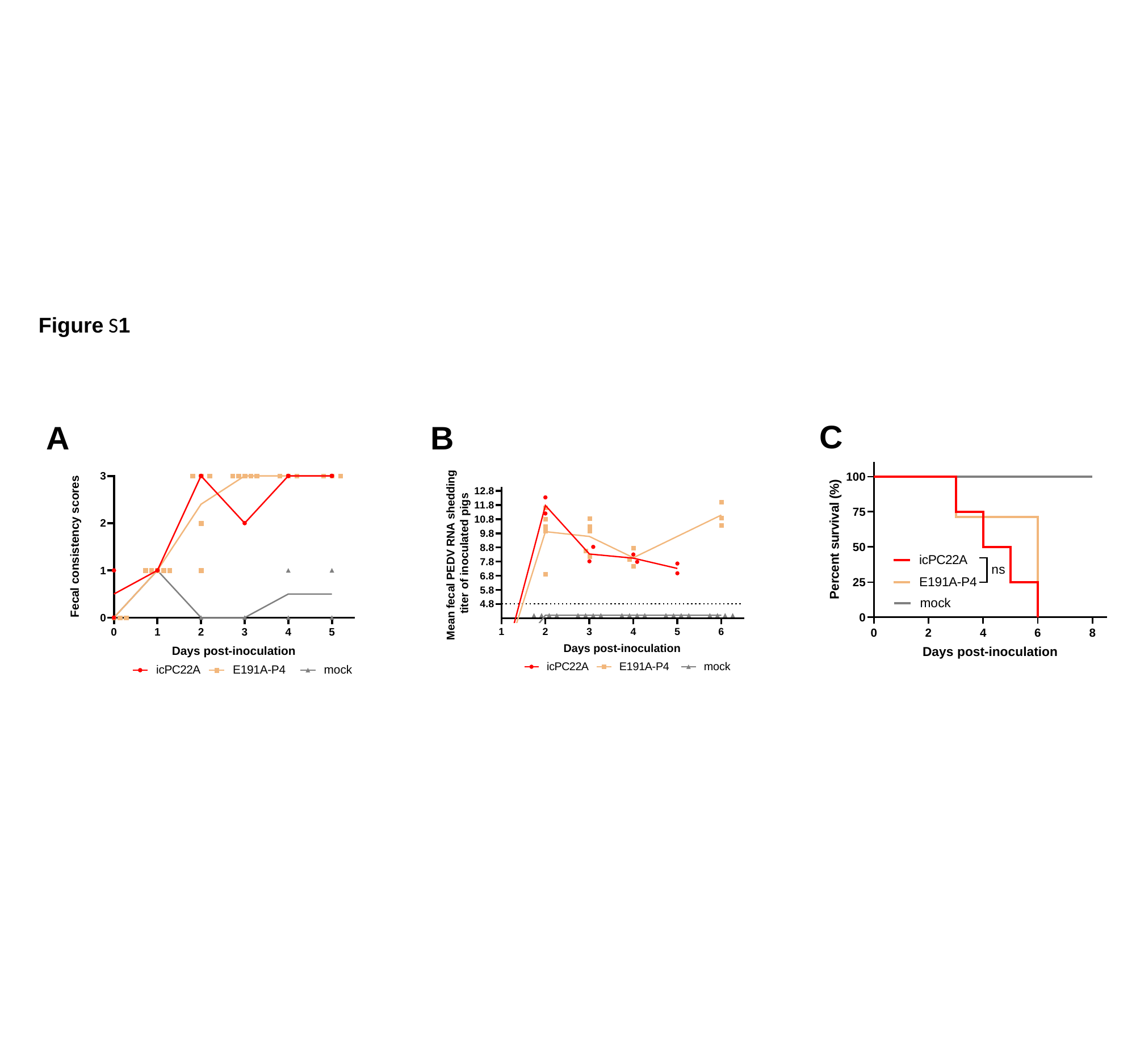

Figure S1

Supplement: Supplementary file 1 — Additional file 1: Figure S1. Pathogenicity of the E191A-P4 in Gn piglets that were initially inoculated with E191A-P1 but did not shed virus by 3 dpi and re-inoculated with E191A-P4 at 3 dpi. (A) Fecal consistency scores of pigs after the E191A-P4 inoculation. Fecal consistency was scored as follows: 0, solid, 1, pasty, 2, semiliquid; and 3, liquid. Scores of ≥ 2 and 3 were considered diarrhea and severe diarrhea, respectively. Each dot represents the score of an individual pig; each line indicates the mean scores of a group. (B) PEDV RNA (N gene) shedding titers in rectal swabs after inoculation. Each line indicates the mean values of a group. The dash line at 4.8 log10 copies/mL indicates the detection limit. (C) Survival curves of pigs after inoculated with E191A-P4. [file 13578_2021_598_MOESM1_ESM.pptx]
